# Eveningness and Procrastination: An Exploration of Relationships with Mind Wandering, Sleep Quality, Self-Control, and Depression

**DOI:** 10.3390/ejihpe15050079

**Published:** 2025-05-13

**Authors:** Richard Carciofo, Rebecca Y. M. Cheung

**Affiliations:** 1 School of Psychology and Clinical Language Sciences, University of Reading, Reading RG6 6AH, UK; r.g.carciofo@reading.ac.uk; 2 Department of Educational Studies, Academy of Future Education, Xi’an Jiaotong-Liverpool University, 111 Ren’ai Road, Suzhou Industrial Park, Suzhou 215123, China

**Keywords:** chronotype, depression, mind wandering, morningness–eveningness, procrastination, self-control, sleep quality

## Abstract

While morningness (a preference for rising earlier in the day) is associated with positive affect and life satisfaction, eveningness is correlated with negative emotionality, poor sleep, less self-control, and more procrastination. The current study investigated inter-relationships between morningness–eveningness; bedtime, academic, and exercise procrastination; mind wandering; sleep quality; self-control; and depressive symptoms. An online survey including questionnaire measures of these variables was completed by 306 university students (aged 18–51 years; mean = 20.36, SD = 4.001; 34 male). Morningness correlated with more self-control and better sleep quality—eveningness correlated with more bedtime, academic, and exercise procrastination; depressive symptoms; and mind wandering. All forms of procrastination negatively correlated with self-control and sleep quality, and positively correlated with depressive symptoms and mind wandering, although more strongly with spontaneous than deliberate mind wandering. Mediation effects were found—bedtime procrastination (BP) between eveningness and spontaneous mind wandering (MW); spontaneous MW between BP and sleep quality; sleep quality between BP and depressive symptoms; self-control between depressive symptoms and academic procrastination. A path model of these inter-relationships was developed. This study adds to a growing body of research indicating that interventions to reduce bedtime procrastination may bring about improvements in wellbeing and academic achievement.

## 1. Introduction

Morningness–eveningness preference refers to individual differences in preferred times for getting up and going to bed, the times for being more alert, and preferred times for activity (Adan et al., 2012). Morningness (a preference for rising and being more active earlier in the day) is associated with indices of wellbeing, positive affect, and more life satisfaction (Biss & Hasher, 2012; Hasan et al., 2022; Howell et al., 2008; Randler, 2008), while eveningness is associated with negative emotionality and indices of poor wellbeing, including anxiety (Cox & Olatunji, 2019), stress (Roeser et al., 2012), negative mood (Díaz-Morales et al., 2015), and depression (Au & Reece, 2017; Kivelä et al., 2018; Merikanto et al., 2013). Eveningness is also associated with more use of substances, including alcohol, nicotine, energy drinks, and caffeine (Arrona-Palacios et al., 2020; Suh et al., 2017; Siudej & Malinowska-Borowska, 2021; Wittmann et al., 2006), more electronic screen time at night (Shimura et al., 2018), poorer sleep quality (Bakotic et al., 2017), and poorer academic achievement (A. N. Önder et al., 2023; Scherrer & Preckel, 2021; Zajenkowski et al., 2024).

Furthermore, eveningness is associated with having less effective self-regulation, less self-control, less conscientiousness, and more present-hedonistic time perspective, while morningness is associated with more self-control, more conscientiousness, and more future time perspective (Digdon & Howell, 2008; Hairston & Shpitalni, 2016; Lipnevich et al., 2017; Stolarski et al., 2013). Consistent with this, eveningness has also been associated with more procrastination (delaying intended activities despite potential negative consequences), including general procrastination (Díaz-Morales et al., 2008; Digdon & Howell, 2008), academic procrastination (A. N. Önder et al., 2023), and bedtime procrastination (Kadzikowska-Wrzosek, 2018; Meng et al., 2021; Pu et al., 2022; Zhu et al., 2023). Like eveningness, procrastination is associated with depressive symptoms (Cui et al., 2021; Hairston & Shpitalni, 2016). Bedtime procrastination is associated with poor self-regulation, depression, insufficient sleep, poor sleep quality, daytime fatigue, and so also the related health implications (Kroese et al., 2014; Kadzikowska-Wrzosek, 2018; Pu et al., 2022; Zhu et al., 2023). Academic procrastination is associated with poorer academic achievement and more depressive symptoms (A. N. Önder et al., 2023; Kınık & Odacı, 2020). Symptoms of depression, such as a lack of interest in activities, fatigue, indecisiveness, negative views of the self, the world, and the future, in addition to low mood and a lack of motivation, may reduce the ability for self-control and increase the likelihood of academic procrastination (Kınık & Odacı, 2020; Remster, 2014). Increased mind wandering (task-unrelated thought) is a mutual correlate of eveningness and procrastination, while greater mindfulness is associated with more morningness and less procrastination (Carciofo et al., 2014; Cheung & Ng, 2019; Kum et al., 2024; Teoh et al., 2021).

The inter-connectedness of these variables is indicated by findings such as that bedtime procrastination mediates the relationship between eveningness and sleep quality (Zhu et al., 2023), and poor sleep quality mediates between eveningness and depression (Bakotic et al., 2017). Also, a cross-lagged panel analysis revealed that procrastination longitudinally predicted mind wandering, but not vice versa (Kum et al., 2024), suggesting the possibility that mind wandering may mediate between bedtime procrastination and sleep quality. Furthermore, Cui et al. (2021) found in another cross-lagged panel analysis that bedtime procrastination predicted later depressive symptoms, but not vice versa, and they suggest that sleep quality may mediate between bedtime procrastination and depression. More depression is longitudinally associated (at one year) with subsequent lower self-control, with depressive symptoms potentially contributing to a depletion of self-control resources (Remster, 2014).

### The Present Study

Following on from the reviewed research, the current study aimed to further understand the relationships between morningness–eveningness, procrastination, mind wandering, sleep quality, depressive symptoms, and self-control. While a host of studies have examined the links between some of the study variables (e.g., Bakotic et al., 2017; Cui et al., 2021), to our knowledge, few if any have investigated the relationships across all of the variables. The present study aims to provide new findings by addressing the following research questions:

Firstly, is eveningness associated with exercise procrastination? Given that eveningness is associated with other types of procrastination, and that morningness is associated with more physical activity (Suh et al., 2017), more eveningness may be expected to correlate with more exercise procrastination. Secondly, is procrastination (bedtime, academic, and exercise) associated with more spontaneous or deliberate mind wandering? Conceptually, more procrastination may be related to both intentional internal distraction from a task, as one means of delaying task initiation/completion, and also to having a greater propensity for spontaneous/unintended internal distractions which would also delay task initiation/completion, perhaps through losing track of time, a lack of focus on the present, and/or a lack of meta-awareness (Teoh et al., 2021).

Furthermore, the research literature reviewed above suggests several possible mediation effects which were tested: (1) As eveningness predicts bedtime procrastination (Zhu et al., 2023) and procrastination predicts mind wandering (Kum et al., 2024), does bedtime procrastination mediate between eveningness and mind wandering (spontaneous and/or deliberate)? (2) Following on from this, as mind wandering may be one mechanism by which sleep is delayed, does it mediate between bedtime procrastination and sleep quality? (3) As bedtime procrastination mediates between eveningness and sleep quality (Zhu et al., 2023) and poor sleep quality mediates between eveningness and depression (Bakotic et al., 2017), then (as suggested by Cui et al., 2021) does sleep quality mediate between bedtime procrastination and depression? (4) As the symptoms of depression (reduced motivation, fatigue, indecisiveness, etc.) may impair self-control, increasing the likelihood of academic procrastination (Kınık & Odacı, 2020; Remster, 2014), does self-control mediate between depression and academic procrastination? Finally, building on the results of these analyses, a path model was developed for the inter-relationships between morningness–eveningness, procrastination, mind wandering, sleep quality, depressive symptoms, and self-control.

## 2. Method

### 2.1. Sample

A sample of university students, aged at least 18 years old, were recruited to complete an online survey in return for course credits. Three items were included throughout the survey as checks on attention/careful responding. Each of these items directed the respondent to answer a particular option for that item; data for respondents who failed to do this for at least 2/3 of the items were excluded from the analysis. In total, 321 participants began the survey; after removing duplicates and incomplete survey records, and respondents who failed the validity check, the final sample consisted of *N* = 306, mean age = 20.36 years (*SD* = 4.001; range = 18–51), with 34 self-identified as men (*M* = 20.65, *SD* = 2.460), 265 as women (*M* = 20.31, *SD* = 4.194), and 7 as ‘other’ (*M* = 20.71, *SD* = 2.430). Male–female age difference, *t* = 0.459, *p* = 0.647. Male–other age difference, *t* = −0.066, *p* = 0.948. Female–other age difference, *t* = −0.254, *p* = 0.800. Self-reported ethnicity was: 62.7% White, 19.6% Asian, 5.2% Black, 6.9% Mixed, and 5.6% Other.

The online survey began with a briefing of the study aims which also stated that participation was voluntary and could be withdrawn at any time, and that data would be de-identified. Electronic consent was obtained before beginning the survey questions. Ethics approval for the research protocol was provided by the University of Reading School of Psychology and Clinical Language Sciences Research Ethics Committee (research number: 2024-004-RC).

### 2.2. Materials

*The reduced Morningness-Eveningness Questionnaire* (rMEQ; Adan & Almirall, 1991) is comprised of five items from the *Morningness-Eveningness Questionnaire* (Horne & Östberg, 1976), each scored with a 4- or 5-point Likert-type response format. It gives an overall assessment of subjective morningness–eveningness preferences, with higher total scores indicating more morningness. Sample items include, “At what time in the evening do you feel tired and as a result in need of sleep?” and “At what time of the day do you think that you reach your “feeling best” peak?” The rMEQ correlates well with other morningness–eveningness scales and with objective measures of sleep schedules, such as actigraphy (Thun et al., 2012).

*The Bedtime Procrastination Scale* (BPS; Kroese et al., 2014) has 9 items (four reverse-scored) assessing the frequency of delaying bedtime. Sample items include, “I go to bed later than I had intended” and “I easily get distracted by things when I actually would like to go to bed”. Criterion validity has been indicated in expected associations with self-regulation and sleep sufficiency. Each item is scored from 1 (almost) never to 5 (almost) always, with higher summed scores indicating more procrastination.

*The Academic Procrastination Scale-Short Form* (APS-S; Yockey, 2016) has 5 items assessing procrastination related to academic activities and has shown good internal consistency and convergent validity with other measures of procrastination. Sample items include, “I know I should work on schoolwork, but I just don’t do it” and “I put off projects until the last minute”. Each item was rated from 1 (strongly disagree) to 5 (strongly agree).

*The Procrastination in Exercise Scale* (PiES; Kelly & Walton, 2021) has five items assessing procrastination related to exercise and predicts levels of physical activity. Sample items include, “I delay working out” and “I often find myself feeling behind on my exercise routines”. Each item is assessed from 1 (strongly disagree) to 5 (strongly agree).

*The Self-Control Scale* (Tangney et al., 2004) has 13 items (nine reverse-scored) assessing perceived self-control, and has shown criterion validity and test–retest reliability. Sample items include, “I am good at resisting temptation” and “People would say that I have iron self-discipline”. Each item is scored from 1 (not at all) to 5 (very much).

*The Mind Wandering: Deliberate and Mind Wandering: Spontaneous Scales* (Carriere et al., 2013) contain four items assessing deliberate mind wandering and four items assessing spontaneous mind wandering. Sample items include, “I allow my thoughts to wander on purpose” (deliberate mind wandering) and “I find my thoughts wandering spontaneously” (spontaneous mind wandering). Expected associations with attentional control and good internal consistency have been shown. Each item is scored on a 7-point scale so that higher scores indicate more mind wandering.

*The Single-item Sleep Quality Scale* (Snyder et al., 2018), assesses overall sleep quality over the last seven days, from 0 (terrible) to 10 (excellent), and has shown concurrent validity and good test–retest reliability. The item reads, “During the past 7 days, how would you rate your sleep quality overall?”

Of *The Depression Anxiety Stress Scales* (DASS; Lovibond & Lovibond, 1995), only the 7-item depression subscale was used. Items are scored on a 0–3 scale for the past week; higher scores indicate more depressive symptoms. Sample items include, “I felt that I had nothing to look forward to” and “I couldn’t seem to experience any positive feeling at all”. Convergent and discriminant validity have been shown for the DASS-21 subscales (Henry & Crawford, 2005).

Regarding demographic information., in addition to age, gender, and ethnicity, participants reported whether they had ever been diagnosed with a sleep disorder (yes/no), and whether they had ever been diagnosed with a depressive disorder (yes/no).

### 2.3. Data Analysis

Descriptive statistics were calculated for each scale/subscale—the mean, standard deviation, range, skewness, kurtosis, and Cronbach’s alpha (internal consistency). Pearson correlations were calculated, for which coefficients of 0.10, 0.30, and 0.50 may indicate small, medium, and large effect sizes, respectively, and *N* = 85 may establish medium effect sizes with 80% power at *p* = 0.05 (Cohen, 1992). Previously reported correlations between study variables have been in the range of small to medium/strong (e.g., morningness–eveningness correlations with self-control and bedtime procrastination). The obtained sample of *N* = 306 was sufficient for establishing small–medium correlations of around 0.2 with 80% power at the 5% significance level (https://homepage.univie.ac.at/robin.ristl/samplesize.php?test=correlation (accessed on 7 January 2024)). Bonferroni corrections have not been applied, given disagreement about when and how they should be used, and the increased risk of Type II errors (Nakagawa, 2004). Following previous research (Seli et al., 2019), partial correlations were calculated for deliberate mind wandering when controlling for spontaneous mind wandering and vice versa, to establish unique associations with each. PROCESS (Hayes, 2022) was utilised for mediation analysis—indirect effects and their 95% percentile bootstrap confidence intervals were calculated from 5000 bootstrap samples; significant mediation effects are indicated when confidence intervals exclude zero. IBM Amos (v.28) was utilised to develop a path model. Acceptable model fit was indicated by the following guidelines (Brown, 2006): the Comparative Fit Index (CFI) and the Tucker–Lewis Index (TLI) > 0.90; root mean square error of approximation (RMSEA) < 0.08; standardised root mean square residual (SRMR) < 0.08.

## 3. Results

The descriptive statistics are shown in Table 1. Scores covered the scale ranges and approximated normal distributions with mostly low values of skewness and kurtosis (<1). Internal consistency was good, with values of Cronbach’s alpha all >0.70. Only 8/306 (2.6%) reported ever being diagnosed with a sleep disorder; 59/306 (19.3%) reported ever having been diagnosed with a depressive disorder.

Age significantly correlated with morningness, *r* = 0.146, bedtime procrastination, *r* = −0.135, and academic procrastination, *r* = −0.123 (*ps* < 0.05), while the only significant difference for males/females was for self-control: male mean = 34.74 (SD = 6.802), female mean = 37.57 (SD = 7.958), *t* = −1.985, *p* = 0.048.

### 3.1. Correlation Analysis

Correlations between scales are shown in Table 2. More eveningness (lower rMEQ scores) had significant small to strong correlations with deliberate and spontaneous mind wandering (MW); bedtime, exercise, and academic procrastination; and depressive symptoms. Morningness had significant small–medium correlations with sleep quality and self-control. Both deliberate and spontaneous MW had significant positive correlations with each type of procrastination, and negative correlations with self-control; the coefficients were all stronger for spontaneous MW than deliberate MW. Depressive symptoms had small–medium significant positive correlations with each type of procrastination, and spontaneous MW. Sleep quality had significant small–medium negative correlations with each type of procrastination, spontaneous MW, and depressive symptoms. Self-control had medium–strong negative correlations with procrastination and depressive symptoms, and a positive correlation with sleep quality.

When controlling for spontaneous MW, correlations with deliberate MW were mostly attenuated, and those with bedtime procrastination, exercise procrastination, and self-control became weak and non-significant, while the negative correlation with depressive symptoms became stronger (Table 2). When controlling for deliberate MW, correlations with spontaneous MW mostly showed some attenuation but with no changes in statistical significance at *p* ≤ 0.05.

### 3.2. Mediation Analysis

The first model tested bedtime procrastination (BP) as a mediator between eveningness and mind wandering (MW). With spontaneous MW as the outcome variable, a significant indirect effect was found (Table 3, model 1). The direct effect of eveningness on spontaneous MW was not significant. With deliberate MW as the outcome variable, the indirect effect was not significant (−0.0969; 95% CI = −0.2197/0.0227).

The second model tested MW as a mediator between bedtime procrastination and sleep quality. A significant indirect effect was found for spontaneous MW (Table 3, model 2), but bedtime procrastination retained a significant direct effect on sleep quality. The indirect effect for deliberate MW was not significant (0.0021; 95% CI = −0.0046/0.0100).

The third model tested sleep quality as a mediator between bedtime procrastination and depressive symptoms. A significant indirect effect was found (Table 3, model 3); the direct effect of bedtime procrastination on depressive symptoms was not significant.

The fourth model tested self-control as a mediator between depressive symptoms and academic procrastination. A significant indirect effect was found (Table 3, model 4), and the direct effect from depressive symptoms to academic procrastination was not significant.

**Figure 1:**
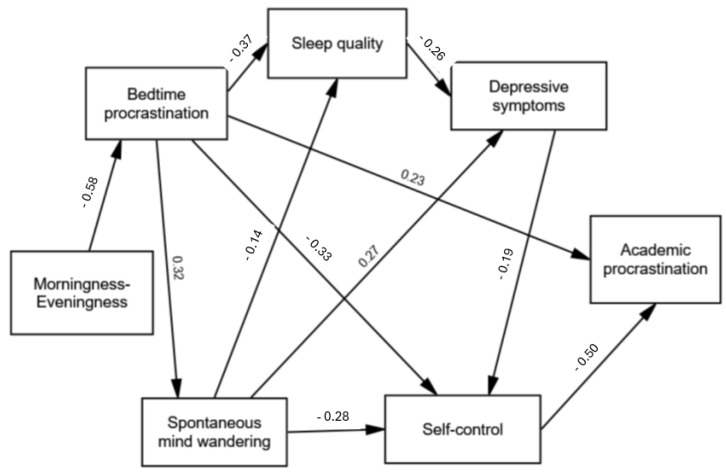
Path model with standardised coefficients.

Finally, building on the results of the mediation analysis, an exploratory (post hoc) path model was developed with morningness–eveningness as the predictor for bedtime procrastination (BP), and paths through spontaneous mind wandering (MW), sleep quality, depressive symptoms, self-control, and then to academic procrastination as the outcome variable. To improve the model fit, changes were made with consideration of the significance of paths and modification indices to guide theoretically meaningful changes to the model. Full details of the development of the model are given in the Supplementary Materials. The final model (Figure 1) showed good fit indices: Chi-square = 13.352; df = 10; *p* = 0.205; CFI = 0.994; TLI = 0.988; RMSEA = 0.033 (90% CI = 0.000/0.075); SRMR = 0.0332. All paths were significant (*ps* < 0.05). The model fit remained good after including age, gender, diagnosis of a sleep disorder, and diagnosis of a depressive disorder as covariates (details in the Supplementary Materials).

## 4. Discussion

The current study aimed at further understanding the association between morningness–eveningness and procrastination, with consideration of relationships with mind wandering, sleep quality, depressive symptoms, and self-control. It was assessed whether the eveningness–procrastination association extends to exercise procrastination, and whether procrastination (bedtime, academic, and exercise) is more strongly associated with spontaneous or deliberate mind wandering. Possible mediation models were developed from the existing research literature, and the findings were integrated into an exploratory path model.

Firstly, correlational analysis replicated previous research findings, including morningness being correlated with more self-control (Digdon & Howell, 2008) and eveningness being correlated with more depressive symptoms (Au & Reece, 2017; Kivelä et al., 2018; Merikanto et al., 2013), poor sleep quality (Bakotic et al., 2017), and mind wandering (Carciofo et al., 2014); self-control was negatively correlated with depressive symptoms (Remster, 2014; Tangney et al., 2004). Furthermore, eveningness was associated with bedtime procrastination (Kadzikowska-Wrzosek, 2018; Meng et al., 2021; Zhu et al., 2023), academic procrastination (A. N. Önder et al., 2023), and also with more exercise procrastination, extending the finding that morningness is associated with more physical activity (Suh et al., 2017). All forms of procrastination were positively correlated with depressive symptoms, and negatively correlated with self-control and sleep quality, consistent with prior findings (Cui et al., 2021; Hairston & Shpitalni, 2016; Kınık & Odacı, 2020; A. N. Önder et al., 2023; Pu et al., 2022).

In addition, all forms of procrastination showed positive correlations with both deliberate and spontaneous mind wandering (MW), indicating that more procrastination may involve both distraction from a task by intentionally switching to unrelated thoughts, and having a greater propensity for spontaneous/unintended task-unrelated thoughts. However, the correlations were all stronger for spontaneous MW and, when controlling for spontaneous MW, the correlations with deliberate MW became weak and non-significant. When controlling for deliberate MW, there was less attenuation of correlations with spontaneous MW, which remained statistically significant.

Mediation analysis tested models derived from previous research (Bakotic et al., 2017; Cui et al., 2021; Kınık & Odacı, 2020; Kum et al., 2024; Remster, 2014; Zhu et al., 2023). Firstly, bedtime procrastination (BP) was found to mediate between eveningness and mind wandering (MW), but this was only significant for spontaneous MW, not for deliberate MW; the direct effect of eveningness on spontaneous MW was not significant. Secondly, MW mediated between bedtime procrastination and sleep quality. Again, this was only significant for spontaneous MW; bedtime procrastination retained a significant direct effect on sleep quality. Thirdly, sleep quality mediated between bedtime procrastination and depressive symptoms; the direct effect of bedtime procrastination on depressive symptoms was not significant. And finally, self-control mediated between depressive symptoms and academic procrastination, with the direct effect from depressive symptoms to academic procrastination not being significant.

Building on these results, a path model was developed for morningness–eveningness predicting bedtime procrastination, with paths from bedtime procrastination to sleep quality, spontaneous MW, self-control, and academic procrastination; sleep quality to depressive symptoms; spontaneous MW to sleep quality, depressive symptoms, and self-control; depressive symptoms to self-control; and, self-control to academic procrastination. The model showed good fit, including after controlling for covariates.

While this model should be further tested, it presents a coherent integration of established findings. Eveningness predicts bedtime procrastination (Kadzikowska-Wrzosek, 2018; Meng et al., 2021; Zhu et al., 2023). Evening-types may delay tasks, such as academic work, until the afternoon/evening when they are more alert/can work more effectively (Díaz-Morales et al., 2008; A. N. Önder et al., 2023), although they may also experience competing distractions, with, for example, opportunities for social activities in the evening. Also, as evening-types do not feel tired until later in the evening, they may be more likely to initiate activities to engage their time, including the use of electronic devices at night (Shimura et al., 2018) which may then further delay going to bed.

Furthermore, consistent with longitudinal evidence that general procrastination predicts later mind wandering (Kum et al., 2024), the current results indicate that bedtime procrastination may increase the likelihood of spontaneous MW. In the case of bedtime procrastination, a theoretical basis for this may be MW episodes becoming more likely to occur as executive control failures become more frequent due to increasing sleepiness (Carciofo et al., 2014; McVay & Kane, 2010). More mind wandering may also predict subsequent difficulty with falling asleep (Ottaviani & Couyoumdjian, 2013), perhaps due to intrusive mind wandering episodes related to ongoing current concerns (McVay & Kane, 2010). In addition, sleep quality may mediate between mind wandering and negative affect (Carciofo et al., 2014).

The well-established effects of poor sleep quality/lack of sleep include negative emotionality and depression, and impaired cognitive functioning (Alvaro et al., 2013; Augner, 2011; Åkerstedt, 2007; Alapin et al., 2000; Sun et al., 2018). In addition to more bedtime procrastination and the associated impacts on sleep, evening-types may experience social jetlag (rising earlier than desired due to work, study, etc.; Wittmann et al., 2006), thereby rising at a time closer to the nadir of their circadian core body temperature rhythm, increasing the likelihood of sleep inertia (Scheer et al., 2008), which also adversely impacts cognitive functioning and task performance (Jewett et al., 1999; Lundholm et al., 2021; Occhionero et al., 2021). Theoretically, these negative impacts on cognitive functioning may include fewer cognitive resources being available for self-regulation; the related experience of depressive symptoms may also contribute to the depletion of self-control resources (Remster, 2014). Consistent with this possibility, it has been found that the positive association between morningness and self-control is mediated by experiencing less social jetlag (Wang & Hu, 2016). Furthermore, the influence of conscientiousness on task engagement/persistence may be weaker when experiencing ‘self-regulatory fatigue’ subsequent to undertaking a task involving more self-regulatory effort (Nes et al., 2011). As a reduction in cognitive resources limits the ability for future-focused/prospective thought (Smallwood et al., 2009), then this may also provide a theoretical basis for an increased likelihood of procrastination, due to having fewer resources available to prospectively plan for/engage with tasks.

Although the current study did not include a measure of academic achievement, procrastination is associated with poorer academic achievement (Kim & Seo, 2015; A. N. Önder et al., 2023), as are eveningness (İ. Önder et al., 2014; Scherrer & Preckel, 2021), social jetlag (Borgio & Louzada, 2021), depression, reduced self-regulation, and lower conscientiousness (Richardson et al., 2012). Longitudinal research has shown that reduced conscientiousness may mediate between social jetlag and poorer academic achievement (Borgio & Louzada, 2021), and that academic procrastination may mediate between conscientiousness and academic achievement (Sparfeldt & Schwabe, 2024). Thus, it would be informative for longitudinal research to test if changes in eveningness predict subsequent changes in these associated variables. For instance, a shift towards more eveningness occurs in adolescence (Adan et al., 2012), so it may be investigated whether greater shifts towards evening chronotype longitudinally predict more bedtime procrastination, more social jetlag, lower conscientiousness, more academic procrastination and lower achievement. Such longitudinal evidence may support suggestions for the development of interventions to reduce bedtime procrastination (Cui et al., 2021), such as using phones to prompt reminders about sleeping, and using educational interventions to improve sleep hygiene awareness, which may influence the relationship between chronotype and bedtime procrastination (Zhu et al., 2023). In addition, as adolescents whose bedtimes are set by their parents may show an earlier chronotype (Randler et al., 2009), educational interventions may include how consistent, appropriate bedtimes may potentially influence chronotype and bedtime procrastination.

### Limitations and Future Directions

The findings should be interpreted in light of several limitations. Firstly, this study utilised self-report measures, which could result in method bias (Podsakoff et al., 2012). Future research should adopt a multi-method, multi-informant approach to increase the objectivity of evaluating the constructs under study. Second, the present study employed a cross-sectional design. As such, longitudinal research is needed to evaluate the directionality of effects (Maxwell & Cole, 2007). Experimental design is also necessary to draw conclusions on causality and eliminate potential confounds. Third, the present sample consisted primarily of women. Even though the age range was 18–51 years, the average age was 20.36 years (*SD* = 4.001). To increase generalisability, future studies should include a gender-balanced sample with a greater spread in age. Relatedly, our sample was drawn from a university in Southern England and was not representative of the general population of the United Kingdom. Future research involving a larger sample of participants from diverse backgrounds and of varied ages could provide valuable insights into the generalizability of effects, based on greater statistical power. Additionally, understanding the variability across ethnic and cultural groups could provide further insights into between-group similarities and differences.

Furthermore, a single item was used to measure sleep quality (Snyder et al., 2018), thereby precluding a differentiation between different types of quality, such as sleep latency and sleep efficiency (Nelson et al., 2022). A fruitful avenue for future research would be to examine why and how bedtime procrastination is related to different types of sleep quality. The current study used a unidimensional measure of morningness–eveningness, so future studies may include measures of morning affect (sleep inertia) and distinctness (the amplitude of diurnal variations in functioning), to investigate associations with these components of circadian functioning. Also, information may be collected about the activities involved in procrastination, such as schoolwork delaying bedtime, and comparisons may be made between weekdays and weekends, for which the significance of bedtime procrastination and eveningness as predictors of sleep timing may vary (Pu et al., 2022).

Although the present findings can inform existing theories of stress and personality, such as cognitive resource theory (Fiedler & Garcia, 1987) and self-determination theory (Deci & Ryan, 2000), follow-up research is needed to test these, and the theorised resource-depletion basis for some of the observed relationships, and/or to formulate and test new theories.

The associations between morningness–eveningness, procrastination, sleep quality, depressive symptoms, and self-control may be complex, and may involve bidirectional relationships such as have been reported for sleep-related factors and depression (Alvaro et al., 2013; Dinis & Bragança, 2018; Sun et al., 2018), sleep quality and bedtime procrastination (Cui et al., 2021), and depression and chronotype (Haraden et al., 2017). So, alternative models of the relationships between these variables may also be investigated, as may additional correlates such as time perspective; notably, Meng et al. (2021) reported that more future time perspective was a mediator between more morningness and less bedtime procrastination. Taken together, although the current path model should be further tested, it presents a coherent integration of established findings. The results provide a coherent picture of the relationships between a set of interrelated variables.

## 5. Conclusions

The current study replicated the correlations between more eveningness and more bedtime and academic procrastination, and also found that eveningness is associated with more exercise procrastination, and that procrastination is more strongly associated with spontaneous mind wandering than with deliberate mind wandering. Bedtime procrastination mediated between eveningness and spontaneous mind wandering; spontaneous mind wandering mediated between bedtime procrastination and sleep quality; sleep quality mediated between bedtime procrastination and depressive symptoms; and self-control mediated between depressive symptoms and academic procrastination. The current results add to other evidence that indicates that interventions to address bedtime procrastination may benefit wellbeing and academic achievement.

## Figures and Tables

**Table 1:** Descriptive statistics.

	Range (Possible)	Mean	Standard Deviation	Skewness	Kurtosis	Cronbach’s Alpha
Morningness–Eveningness	4–23 (4–25)	11.69	3.644	0.390	0.053	0.702
Deliberate Mind Wandering	4–28 (4–28)	18.69	5.332	−0.235	−0.626	0.847
Spontaneous Mind Wandering	5–28 (4–28)	19.33	5.068	−0.561	−0.102	0.836
Bedtime Procrastination	11–45 (9–45)	32.47	6.788	−0.890	0.428	0.867
Exercise Procrastination	6–30 (6–30)	20.58	6.442	−0.456	−0.557	0.947
Academic Procrastination	5–25 (5–25)	17.37	5.316	−0.326	−0.825	0.919
Depressive symptoms	0–21 (0–21)	6.62	5.002	0.752	−0.009	0.908
Self-Control	19–62 (13–65)	37.13	7.970	0.475	−0.015	0.827
Sleep Quality	0–9 (0–10)	5.01	2.226	−0.045	−1.004	-

*N* = 306.

**Table 2:** Zero-order Pearson correlations of the study variables.

	MW-S	M-E	BP	EP	AP	Depressive Symptoms	Sleep Quality	Self-Control
Deliberate MW	0.383 ***	−0.185 **	0.183 **	0.142 *	0.252 ***	−0.032	−0.042	−0.246 ***
*MW-D controlling for MW-S*	-	−0.113 *	*0.070*	*0.028*	0.128 *	−0.186 **	*0.064*	*−0.090*
Spontaneous MW	-	−0.217 ***	0.317 ***	0.306 ***	0.373 ***	0.338 ***	−0.258 ***	−0.450 ***
*MW-S controlling for MW-D*	-	−0.161 **	0.272 ***	0.275 ***	0.309 ***	0.379 ***	−0.262 ***	−0.397 ***
Morningness–Eveningness		-	−0.584 ***	−0.359 ***	−0.370 ***	−0.168 **	0.251 ***	0.357 ***
Bedtime Procrastination			-	0.326 ***	0.464 ***	0.206 ***	−0.415 ***	−0.462 ***
Exercise Procrastination				-	0.360 ***	0.232 ***	−0.143 *	−0.358 ***
Academic Procrastination					-	0.237 ***	−0.286 ***	−0.610 ***
Depressive symptoms							−0.333 ***	−0.352 ***
Sleep Quality							-	0.251 ***

*N* = 306. * *p* ≤ 0.05; ** *p* ≤ 0.01; *** *p* ≤ 0.001. MW-S = spontaneous mind wandering; MW-D = deliberate mind wandering; M-E = morningness–eveningness; BP = bedtime procrastination; EP = exercise procrastination; AP = academic procrastination.

**Table 3:** Mediation analysis.

**Regression Model 1: Criterion = Spontaneous Mind Wandering**	**Predictor: Morningness–Eveningness *β***	**Mediator: Bedtime Procrastination *β***	**Unstandardised Indirect Effect (95% CI) [Standardised Indirect Effect (95% CI)]**
*R* = 0.3196, *R*^2^ = 0.1021, *F*(2, 303) = 17.2331 ***	−0.0487	0.2887 ***	−0.2343 (−0.3629/−0.1138) [−0.1685 (−0.2567/−0.0824)]
**Regression Model 2: Criterion = Sleep Quality**	**Predictor: Bedtime Procrastination *β***	**Mediator: Spontaneous Mind Wandering *β***	**Unstandardised Indirect Effect (95% CI) [Standardised Indirect Effect (95% CI)]**
*R* = 0.4361, *R*^2^ = 0.1902, *F*(2, 303) = 35.5776 ***	−0.3711 ***	−0.1399 *	−0.0145 (−0.0277/−0.0027) [−0.0444 (−0.0837/−0.0081)]
**Regression Model 3: Criterion = Depressive Symptoms**	**Predictor: Bedtime Procrastination *β***	**Mediator: Sleep Quality *β***	**Unstandardised Indirect Effect (95% CI) [Standardised Indirect Effect (95% CI)]**
*R* = 0.3416, *R*^2^ = 0.1167 *F*(2, 303) = 20.0181 ***	0.0818	−0.2994 ***	0.0917 (0.0504/0.1379) [0.1244 (0.0679/0.1859)]
**Regression Model 4: Criterion = Academic Procrastination**	**Predictor: Depressive Symptoms *β***	**Mediator: Self-Control *β***	**Unstandardised Indirect Effect (95% CI) [Standardised Indirect Effect (95% CI)]**
*R* = 0.6109, *R*^2^ = 0.3732 *F*(2, 303) = 90.2149 ***	0.0255	−0.6015 ***	0.2252 (0.1527/0.3019) [0.2119 (0.1447/0.2782)]

*N* = 306. * *p* ≤ 0.05; *** *p* ≤ 0.001.

## Data Availability

The data that support the findings of this study are available at: https://doi.org/10.17864/1947.001418.

## Supplementary Materials

The following supporting information can be downloaded at: https://www.mdpi.com/article/10.3390/ejihpe15050079/s1, Figure S1: Final path model, with standardised coefficients.
