# Supplementary material for: Eveningness and Procrastination: An Exploration of Relationships with Mind Wandering, Sleep Quality, Self-Control, and Depression
Source: Eur J Investig Health Psychol Educ. 2025 May 13;15(5):79. doi: 10.3390/ejihpe15050079 (PMC12110641; doi:10.3390/ejihpe15050079)
Supplement: Supplementary file 1 [file ejihpe-15-00079-s001.zip › ejihpe-3524214-supplementary.pdf]

Eveningness and Procrastination: An Exploration of Relationships with Mind Wandering, Sleep Quality, Self-Control, and Depression

Richard Carciofo and Rebecca Y. M. Cheung

Supplementary materials

Exploratory path model

The initial model had the following paths: morningness-eveningness as the predictor for bedtime procrastination (BP); paths from BP to spontaneous mind wandering (MW) and sleep quality; a path from spontaneous MW to sleep quality; a path from sleep quality to depressive symptoms; a path from depressive symptoms to self-control; and a finally a path from self-control to academic procrastination. Although all paths were significant (*ps* < 0.05), this model did not show good fit: Chi-square = 148.250, df = 14, p = 0.000; CFI = 0.794; TLI = 0.646; RMSEA = 0.177; SRMR = 0.1922.

The largest modification index was for a path from bedtime procrastination to self-control. After adding this path (model 2), again all paths were significant (*ps* < 0.05), but model fit was not acceptable: Chi-square = 87.531; df = 13, p = 0.000; CFI = 0.869; TLI = 0.789; RMSEA = 0.137; SRMR = 0.1123.

The largest modification index was for a path from spontaneous MW to self-control. After adding this path (model 3), again all paths were significant (*ps* < 0.05), but fit statistics were mostly not acceptable: Chi-square = 58.789, df = 12, p = 0.000; CFI = 0.918; TLI = 0.856; RMSEA = 0.113; SRMR = 0.0866.

The largest modification index was for a path from spontaneous MW to depressive symptoms. After adding this path (model 4), all paths were significant and model fit statistics were mostly acceptable: Chi-square = 34.574; df = 11; p = 0.000; CFI = 0.959; TLI = 0.921; RMSEA = 0.084 (90% CI = 0.053/0.116); SRMR = 0.0636.

The largest modification index was for a path from bedtime procrastination to academic procrastination. After adding this path (model 5; Figure S1), all paths were significant and model fit statistics were good: Chi-square = 13.352; df = 10, p = 0.205; CFI = 0.994; TLI = 0.988; RMSEA = 0.033 (90% CI = 0.000/0.075); SRMR = 0.0332. No modification indices were suggested.

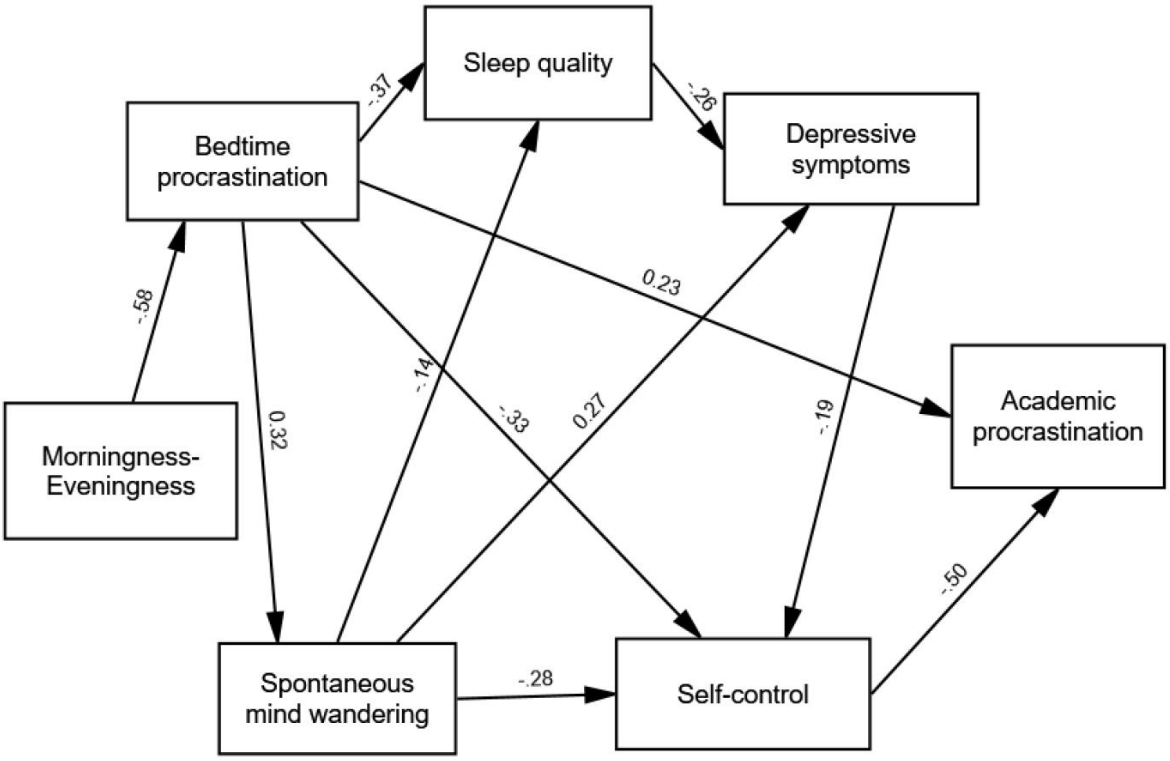

Figure S1. Final path model, with standardised coefficients.

When age, diagnosis of depression, and diagnosis of sleep disorder were added as covariates to the model all paths were significant (*ps* < 0.05), and model fit statistics were good: Chi-square = 13.237, df = 10, $p = 0.211$; CFI = 0.995; TLI = 0.977; RMSEA = 0.033 (90% CI = 0.000/0.074); SRMR = 0.0225. No modification indices were suggested.

When age, diagnosis of depression, diagnosis of sleep disorder, and gender (men/women) were added as covariates to the model ($N = 299$), model fit statistics were good: Chi-square = 14.074, df = 10, $p = 0.170$; CFI = 0.993; TLI = 0.962; RMSEA = 0.037 (90% CI = 0.000/0.078); SRMR = 0.0217. All paths were significant (*ps* < 0.05), except for that from spontaneous MW to sleep quality, for which $p = 0.061$. No modification indices were suggested.
